# Natural phenolic metabolites with anti-angiogenic properties – a review from the chemical point of view

**DOI:** 10.3762/bjoc.11.28

**Published:** 2015-02-16

**Authors:** Qiu Sun, Jörg Heilmann, Burkhard König

**Affiliations:** 1 Institute of Organic Chemistry, University of Regensburg, Universitätsstr. 31, 93053 Regensburg, Germany; 2 Institute of Pharmacy, University of Regensburg, Universitätsstr. 31, 93053 Regensburg, Germany

**Keywords:** angiogenesis, natural phenolic compounds, structure–activity relationship, synthesis

## Abstract

Considering the many secondary natural metabolites available from plants, phenolic compounds play a particularly important role in human health as they occur in significant amounts in many fruits, vegetables and medicinal plants. In this review natural phenolic compounds of plant origin with significant anti-angiogenic properties are discussed. Thirteen representatives from eight different natural or natural-like phenolic subclasses are presented with an emphasis on their synthesis and methods to modify the parent compounds. When available, the consequence of structural variation on the pharmacological activity of the molecules is described.

## Introduction

The term angiogenesis is commonly used to describe the biological process of blood vessel growth. Nevertheless, it should be more precisely defined as the formation of new blood vessels from preexisting ones. Under physiological conditions, angiogenesis is vital for foetal development, tissue regeneration and wound healing. From a pathophysiologic perspective, massive vascular growth or abnormal shape formation promotes many ailments including cancer, inflammation, and eye illnesses. Additionally, inadequate vessel preservation or growth leads to ischemia causing myocardial infarction, stroke, and neurodegenerative or obesity-associated conditions [1–2].

The generation of new blood vessels is based on a strictly controlled balance between various soluble and membrane-bound factors showing either anti- or pro-angiogenic activity, which are embedded together with enzymes and signaling molecules (Table 1) into a very complex network of signaling pathways [3–4]. For example, in the case of cancer development, the growing tumor disturbs the angiogenic balance in tissue and induces the secretion of pro-angiogenic factors either by the tumor cells themselves or by cells of the tumor microenvironment. After the tumor reaches a diameter of 1–2 mm, the tumor cells located far away from blood vessels undergo apoptosis or necrosis resulting from the lack of oxygen and nutrients. At that point, the tumor cells express pro-angiogenic factors including growth factors such as the vascular endothelial growth factor (VEGF) and fibroblast growth factor (FGF) and enzymes such as cyclooxygenase 2 (COX-2) and protein kinase A (PKA) as well as signaling molecules such as integrins. The evoked cascade effect subsequently induces the formation of new blood vessels that quickly connect to preexisting blood vessels, thus providing a sufficient supply of nutrients for tumor survival [5]. In addition, the new blood vessels allow the cancer cells to spread from a parent location to other areas causing metastases. Nevertheless, the morphology and pathophysiology of these blood vessels differs significantly from physiological ones as they are less effective and show a lower state of organization and control [3]. Since the discovery of the mechanism of angiogenesis and its crucial role in tumor development, various therapies targeted at interfering with this process were investigated [6]. The favored clinical targets are the VEGF receptors, which have led to the development and approval of monoclonal antibodies against VEGF and VEGF receptor tyrosine kinase inhibitors [3]. However, the existing therapy options with antibodies and VEGF receptor inhibitors showed several clinical limitations, making the search for further clinically relevant targets and other drugs necessary to combat tumor-related angiogenesis.

**Table 1:** Brief overview of the endogenous modulators involved in angiogenic processes.

Group name	Modulators (abbreviation)

Growth factors	Vascular endothelial growth factor (VEGF) Fibroblast growth factor (FGF) Platelet-derived growth factor (PDGF) Transforming growth factor β1 (TGF-β1) Angiopoetins (Ang)
Adhesion molecules	Integrins Cadherins
Proteinases	Matrix metalloproteinases (MMPs) Urokinase plasminogen activator (uPA)
Extracellular matrix proteins	Fibronectin Collagens
Transcription factors	Nuclear factor (erythroid-derived 2)-related factor (Nrf2) Nuclear factor “kappa-light-chain-enhancer” of activated B-cells (NF-κB) Activator protein (AP-1) Hypoxia inducible factor (HIF)
Other signaling molecules	Mammalian target of rapamycin (mTOR)
Other enzymes	Mitogen-activated protein kinases (MAP-kinases) Proteinkinases A and C (PKA and PKC) Proteinkinase BAkt Cyclooxygenase 2 (COX-2) Nitric oxide synthase (NOS)

Secondary natural metabolites have also been identified as attractive candidates for the therapy of pathologically-induced angiogenesis [7–8]. Among such natural products, phenolic or polyphenolic compounds with anti-angiogenic properties have been investigated and the opinion regarding their potential pharmacological impact has changed over the years. While the pharmacological activity of polyphenols was previously considered as unspecific, more recently, observations of a specific interference with biological mechanisms at the molecular level have significantly increased. Especially in the fields of anti-inflammatory activity, chemoprevention and cytoprotection, natural phenolic metabolites such as flavonoids, caffeic acid derivatives and diarylheptanoids were found to have a pleiotropic influence on cellular signaling (e.g., by the inhibition of transcription factors such as NF-κB or Nrf_2_ [9–10], or antioxidative effects [10–11]). Furthermore, polyphenols are found in high concentrations in many fruits and vegetables, resulting in a continuous and long-term intake of such plant phenols. Consequently, their beneficial and protective impact on unbalanced angiogenic processes has been intensively discussed [7–8].

In the last decade, many excellent review articles summarized the biological and pharmacological aspects of anti-angiogenic compounds, including natural compounds containing a phenolic substructure [12–14]. To complement this previously discussed pharmacological point of view, this review focuses on recent reports of anti-angiogenic, natural, phenolic compounds, specifically addressing their chemistry, synthesis and possible structure modifications. Nevertheless, it should be mentioned that the selection of compounds for this review is based on the reports of their pharmacological activity. As the term “anti-angiogenic compound” is not clearly defined and somewhat overly used, it was necessary to unequivocally define this term for this review. The compounds included in the survey were those shown to exhibit anti-angiogenic activity not only in convenient (and often descriptive) cellular in vitro assays (Table 2), but also in molecular in vitro test systems related to the signaling cascades of pathological angiogenesis. Further criterion for inclusion in this survey was the presence of anti-angiogenic activity obtained in ex vivo and in vivo assays (Table 2). Additionally, the observed in vitro activity should be in the lower µM range (or higher), which is high enough to realistically speculate on the anti-angiogenic activity in vivo. As endothelial cells (ECs) have particular significance in angiogenesis, results from cellular assays using primary or immortalized ECs such as human umbilical vein endothelial cells (HUVEC) or human microvascular endothelial cells (HMEC-1) have been given special attention. In contrast, compounds showing in vitro anti-angiogenic activity but also signs of strong unspecific cytotoxic effects in vitro have been excluded. The secondary metabolites discussed include six flavonoids from different subclasses: quercetin, fisetin, epigallocatechin-3-*O*-gallate, xanthohumol, (2*S*)-7,2’,4’-trihydroxy-5-methoxy-8-dimethylallylflavanone and genistein. Other compounds belong to the group of simple phenols (4-hydroxybenzyl alcohol), hydrolysable tannins (ellagic acid), stilbenoids (resveratrol) and diarylheptanoids (curcumin). In addition, acylphloroglucinols, quinolone-substituted phenols and 4-amino-2-sulfanylphenol derivatives are discussed. Some important aspects of the described pharmacological activities of the compounds are summarized in Table 3.

**Table 2:** In vitro, ex vivo and in vivo assays used to evaluate anti-angiogenic activity.^a^

In vitro assays	Assay principles/Detection, read out

Endothelial cell proliferation assays	Cell counting/Increase of cell number Crystal violet/Increase of cell number MTT/Activity of dehydrogenase activity (positively correlated to cell number) Incorporation of [^3^H]thymidine, 5-bromodeoxyuridine into DNA/DNA synthesis (positively correlated to cell number)
Endothelial cell migration assays	Scratch assay/Migration into a denuded area (wound healing)
Endothelial cell differentiation assays	Tube formation, e.g., in Matrigel/Formation of capillary-like tubules
Endothelial-mural cell co-culture assays	Interaction between two cell types (endothelial/mural)/Influence on cell differentiation and proliferation
	
Ex vivo assays	
Aortic ring assay	Aorta of rodents cultured in biological matrices/Outgrowth of branching microvessels
	
In vivo assays	
Chick chorioallantoic membrane assay (CAM)	Extra-embryonic membrane (in ovo, ex ovo)/Growth and branching of blood vessels
Hen's egg test on chorioallantoic membrane (HETCAM)	CAM modification/Growth and branching of blood vessels
Zebrafish	Zebrafish embryos or transgenic zebrafish embryos/Visualization of vascularisation (e.g., with confocal microscopy)
Corneal angiogenesis assay	Corneal injury or implantation of pellets/Vascular response of the cornea
Dorsal air sac model	Ring (filled with tumor cell suspension) implantation (dorsal skin)/Tumor-induced angiogenesis
Mouse models	Genetically engineered mouse models/Xenografts

^a^Molecular or enzyme assays are not included.

**Table 3:** Natural phenolic compounds with anti-angiogenic activity and their evaluated molecular mechanisms of anti-angiogenesis.

Compound name	Mechanisms of anti-angiogenic action

4-Hydroxybenzyl alcohol	Downregulation of VEGF and MMP9 protein expression
Curcumin	Reduction of VEGF expression, inhibition of transcription factors, mTOR pathway and MMP9 protein expression
Ellagic acid	Inhibition of VEGF and PDGF receptor phosphorylation
Resveratrol	Abrogation of VEGF-mediated tyrosine phosphorylation of vascular endothelial (VE)-cadherin, inhibition of VEGF-induced and FGF-2 neovascularization
Quinoline-substituted phenols	Inhibition of VEGF and Transforming Growth Factor-β1 (TGF-β1) expression
4-Amino-2-sulfanylphenol derivatives	Inhibition of protein kinase B/Akt and ABL tyrosine kinase
Natural-like acylphloroglucinol derivatives	Under investigation
(−)-Epigallocatechin gallate (EGCG)	Inhibition of estrogen-stimulated VEGF expression, HIF-1α and NF-κB, inhibition of MMP-2 and MMP-9, inhibition of urokinase plasminogen activator.
Xanthohumol	Inhibition of NF-κB and Akt pathways
Genistein	Inhibition of VEGF and HIF-1α protein expression
Fisetin	Downregulation of VEGF and eNOS expression, inhibition of MMPs activities
Quercetin	Inhibition of the expression of VEGF-2, inhibition of COX-2 and arachidonate 5-lipoxygenase (LOX-5), inhibition of NF-κB, In some cell types it activates angiogenesis.
(2*S*)-7,2’,4’-Trihydroxy-5-methoxy-8-(dimethylallyl)flavanone	Downregulation of reactive oxygen species (ROS) levels and VEGF expression

## Review

### 4-Hydroxybenzyl alcohol

**Figure 1:**
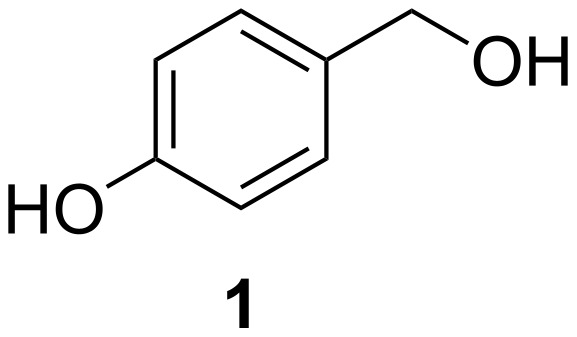
Structure of 4-hydroxybenzyl alcohol (HBA, **1**).

4-Hydroxybenzyl alcohol (HBA, **1**) (Figure 1) is a well-known phenolic compound found in plants and has been isolated from, for example, the flowers of carrot (*Daucus carrota* L., Apiaceae). In 2007, Park and co-workers [15] found no change in the vascular density in the chick chorioallantoic membrane (CAM) assay in the presence of HBA, indicating that HBA had no influence on the growth of the blood vessels. In contrast, the branching pattern of the blood vessels was dose-dependently reduced in the same assay, implying that an inhibition of angiogenesis was plausible. Later, Laschke et al. [16] performed experiments in vitro with an aortic ring assay and in vivo in an endometriosis model as well as the systematic analysis of the mechanism underlying the anti-angiogenic activity of HBA. They found that HBA is capable of inhibiting several steps in the angiogenic mechanism. Western blot analysis showed the downregulation of VEGF and MMP9 protein expression. The effect of HBA was confirmed [17] by mouse dorsal skinfold chamber experiments. The incubation of CT26.WT colon carcinoma cells with HBA showed a dose-dependent decrease in their viability and integrity. In addition, the cell expression of the apoptosis marker, cleaved caspase-3, significantly increased and the expression of vascular endothelial growth factor (VEGF) and matrix metalloproteinase (MMP)-9 decreased compared to the controls. No influence on the normal behavior of the animals was observed. In general, HBA represents an interesting anti-angiogenic agent for the treatment of angiogenic diseases.

### Curcumin

**Figure 2:**
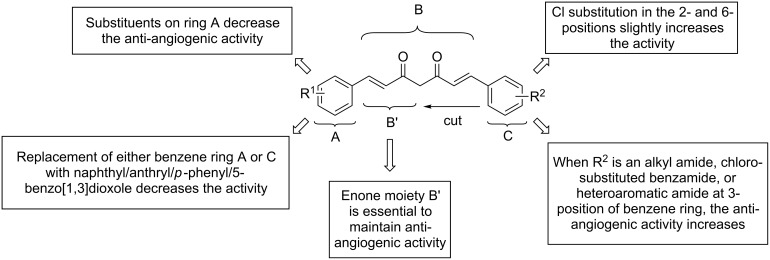
Structure–activity relationship of curcumin analogs.

**Scheme 1:**
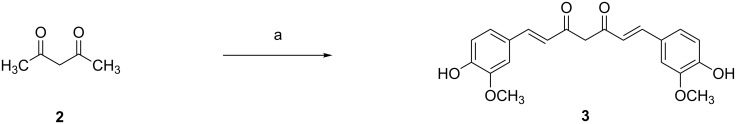
Synthesis of curcumin (**3**). Reagents and conditions: (a) vanillin, 1,2,3,4-tetrahydroquinoline, HOAc, H_3_BO_3_, DMF, Δ, 4 h.

**Figure 3:**
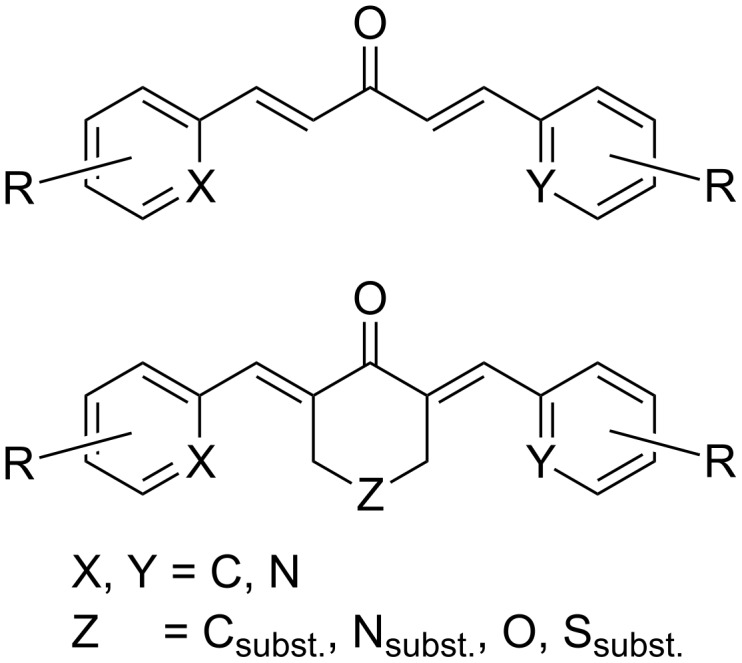
Backbone and substitution of monocarbonyl analogs of curcumin (MACs) showing their structural diversity.

**Scheme 2:**
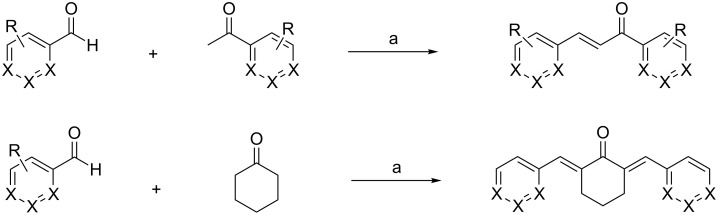
Exemplary synthesis of MAC representatives. Reagents and conditions: (a) 40% KOH, EtOH, 5 °C; stirring 10 h, rt. X = C, N; R = OH, OMe, Cl, F.

Curcumin (**3**) is a natural product isolated from different *Curcuma* species (Zingiberaceae) some of which are used as raw material in the spice turmeric. It has been evaluated as a chemopreventive agent since the early nineties, and in 1998, Arbiser and co-workers [18] found that the compound also demonstrated anti-angiogenic properties in in vitro and in vivo experiments. In the following years, many studies on the anti-angiogenic properties in different tumor cell lines and in animal models were reported [19–22]. They included interactions with the transcription factor NF-κB, mTOR pathway, and reduction of VEGFA and MMP9 expression. Despite its promising pharmacological properties, curcumin suffers from low in vivo bioavailability as a consequence of its low aqueous solubility, poor chemical stability and low absorption. Therefore, many analogs (Figure 2) were synthesized in order to overcome these drawbacks and to enhance the activity. In addition, their structure–activity relationships were studied to gain better insight into the mode of action. The general synthesis of curcumin itself (Scheme 1) [23–24] requires masking of the reactive methylene group of the acetylacetone moiety by formation of a complex with boric oxide, followed by reaction with vanillin. Instead of boric oxide, alkyl borates and boric acid can also be used. The first attempt at modification was to truncate the general structure to either a single enone or dienone system. The latter structure contained a diarylpentanoid moiety instead of the natural diarylheptanoid backbone. In some cases, this was amended by a central ring system and was labeled as monocarbonyl analog of curcumin (MACs, Figure 3). Bowen et al. [25] used the Claisen–Schmidt reaction for the synthesis of these analogs. The C_7_-chain linker between the two aromatic rings was shortened, and a series of compounds (Scheme 2) with different substitutions on the aromatic rings was synthesized to explore the role of stereoelectronic effects. It was demonstrated that these analogs of curcumin showed excellent anti-angiogenic activity with equivalent or superior inhibition to that of the natural parent product. This work was followed by more comprehensive bioactive studies on aromatic enones utilizing the substituted chalcone backbone [26]. The study showed that the presence of the enone moiety played an important role in maintaining the activity in the curcumin analogs. Using this principle, Ahn et al. (2005) [27] left the enone unchanged and prepared various curcumin mimics with asymmetric units bearing alkyl amide, chloro-substituted benzamide, or heteroaromatic amide moieties. These analogs exhibited a stronger anti-angiogenic activity against HUVECs than curcumin. To date, the number of synthesized single enones and MACs have surpassed the 1000 mark.

### Ellagic acid

**Scheme 3:**
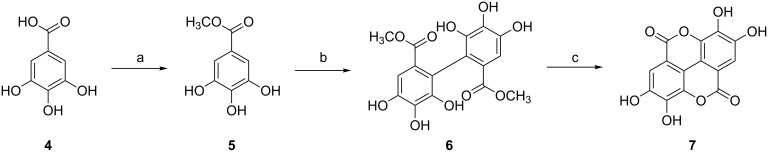
Synthesis of ellagic acid (**7**). Reagents and conditions: (a) H_2_SO_4_, CH_3_OH; (b) (1) *o*-chloranil, Et_2_O, −40 °C; −40 °C → rt, 3 h; (2) Na_2_S_2_O_4_, rt, 30 min; (c) MeOH/H_2_O 1:1, reflux.

Ellagic acid (**7**) is a naturally existing phenolic antioxidant widely found in numerous fruits and vegetables, such as raspberries (*Rubus idaeus* L., Rosaceae), strawberries (*Fragaria* spec. L., Rosaceae) and pomegranates (*Punica granatum* L., Lythraceae). It shows potent antioxidant effects by radical scavenging and the inhibition of lipid peroxidation [28–29]. Ellagic acid is also capable of interfering with some angiogenesis-dependent pathways. It exhibits anticarcinogenic activity through inhibiting of tumor-cell proliferation, migration and induction of apoptosis. In addition, it is a dual inhibitor of the phosphorylation of VEGF and PDGF receptors, thus interrupting the angiogenetic processes required for tumor growth [30]. Recently, it was reported that its anti-angiogenesis mechanism affects the VEGFR-2 signaling pathway by forming hydrogen bonds and aromatic interactions within the ATP-binding region of the VEGFR-2 kinase unit [31]. Shankar and Srivastava et al. [32] treated PANC-1 xenograft mice with ellagic acid (**7**) and measured the expression of protein kinase B (Akt), sonic hedgehog (Shh) and Notch. The results suggested that ellagic acid effectively inhibited human pancreatic cancer growth by suppressing the Akt, Shh and Notch pathways. The preparation of ellagic acid (**7**) can be achieved by the oxidative coupling of gallic acid (Scheme 3) [33]. In the presence of H_2_SO_4_, gallic acid (**4**) was esterified to methyl gallate (**5**). The obtained ester **5** was first oxidatively coupled in the presence of *o*-chloranil, followed by reduction with Na_2_S_2_O_4_ to obtain the hexahydroxy biphenyl **6**. Subsequent lactonization afforded the final product, ellagic acid (**7**), in high yield.

### Resveratrol

**Figure 4:**
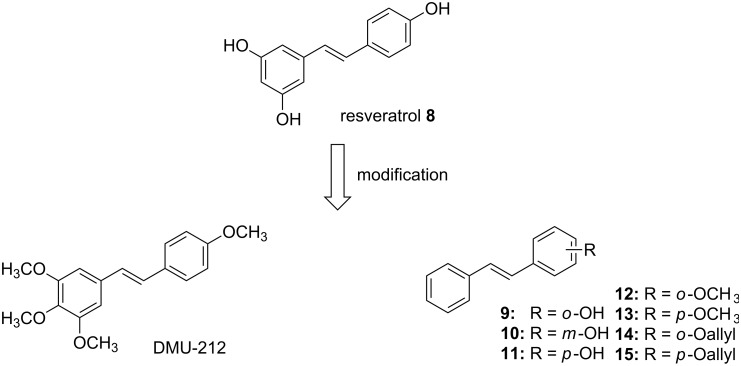
Structure of resveratrol and its analogs.

Resveratrol (**8**) is a natural polyphenol that belongs to the stilbene-type compounds and exists in a large number of plants. It has been primarily extracted from grape (*Vitis vinifera* L., Vitaceae) and mulberry (*Morus* L., Moraceae) fruits (Figure 4) [34]. It has antioxidant effects, anti-estrogenic activity and the ability to reduce the synthesis of hepatic lipids and of eicosanoids. It inhibits platelet aggregation and protects vessels from arteriosclerosis [34–38]. In recent years, it has been reported that resveratrol is sufficiently potent to inhibit VEGF-induced and FGF-2 neovascularization in vivo [39]. It was also shown that resveratrol directly inhibited bovine aorta endothelial cell proliferation, migration and tube formation in vitro [40]. Resveratrol has also been found to effectively interrupt VEGF-mediated tyrosine phosphorylation of vascular endothelial (VE)-cadherin and its complex partner, β-catenin [41]. Unfortunately, resveratrol has a dual effect on cells, depending on the situation and cell type – it can either induce or suppress angiogenic effects [42]. The low oral bioavailability and metabolic stability of resveratrol also limits its application [43]. Therefore, in an attempt to increase its bioavailability and stability, the structure of resveratrol was modified by methylation of the phenol groups [44] and by introduction of other groups on the phenyl ring (compounds **9–15**, Figure 4) [45]. *trans*-3,4,4’,5-Tetramethoxystilbene (DMU-212) has improved pharmacokinetic properties as compared to resveratrol and shows antiproliferation activity in various cancer cells [46–48]. The further investigation of its role in angiogenesis by Dai and Zhang et al. [49] showed that DMU-212, a potential anti-angiogenic agent, inhibits VEGFR2 phosphorylation and thereby acts to suppress the signaling pathways mediated by VEGFR2, inducing apoptosis in endothelial cells.

### Quinoline-substituted phenols

**Scheme 4:**
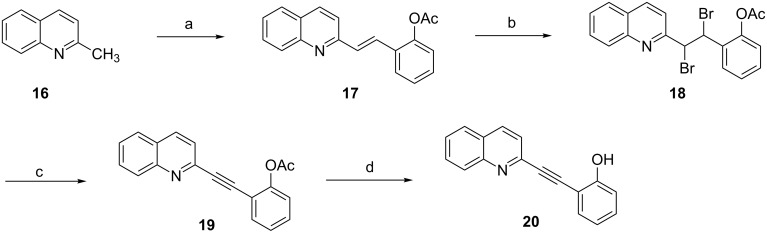
Synthesis of quinolone-substituted phenol **20**. Reagents and conditions: (a) Ac_2_O, 2-hydroxybenzaldehyde, 130 °C; (b) (1) Br_2_, AcOH, (2) Ac_2_O; (c) (1) DBU, THF, (2) Ac_2_O; (d) Na_2_CO_3_, MeOH/THF.

**Scheme 5:**
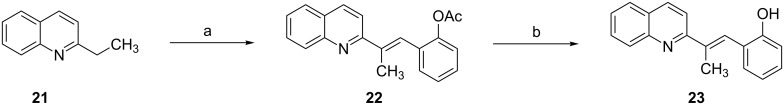
Synthesis of quinolone-substituted phenol **23**. Reagents and conditions: (a) Ac_2_O, 2-hydroxybenzaldehyde; (b) (1) NaOH, EtOH/H_2_O, 100 °C, (2) HCl.

To date, quinolone-substituted phenols (Qsps) have not been reported as natural secondary metabolites, although quinolone and alkylated phenols are common structural elements of secondary plant products. The quinoline skeleton is present in alkaloids derived from tryptophane-like quinine or camptothecine, whereas alkylated phenols with a varying alkyl side chain length are common metabolites from the shikimate pathway. Qsps were reported in a recent patent [50] to be effective for the treatment of angiogenesis-related diseases or disorders. Among other assays, a transgenic line of zebrafish that expresses a fluorescent reporter (EGFP) in vasculature was used in this study to identify anti-angiogenic compounds. Special emphasis was placed on the integrity of the vessels developing in the eyes and in the trunk. Quinoline-substituted phenols were identified as being active based on a significant inhibition of the hyaloid vessel formation in the zebrafish model. The synthesis of two representative compounds, **20** and **23**, of this class are shown in Scheme 4 and Scheme 5, respectively [50].

After condensation of **16** with hydroxybenzaldehyde, product **17** was subsequently brominated, followed by twofold elimination of HBr forming the triple bond. Finally, ester hydrolysis provided compound **20**. Compound **23** was obtained from an analogous condensation product, **22**, by hydrolysis under basic condition.

### 4-Amino-2-sulfanylphenol derivatives

**Figure 5:**
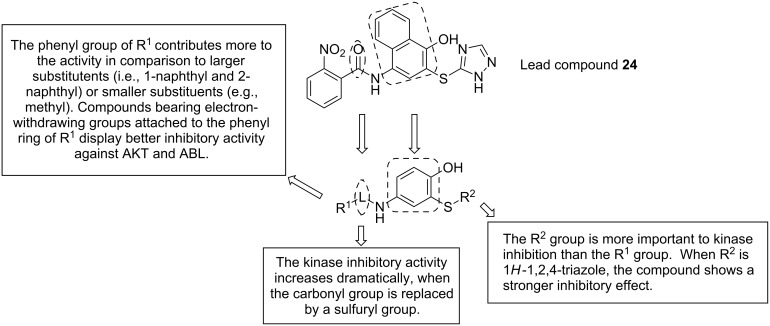
Design of 4-amino-2-sulfanylphenol derivatives and their structure–activity relationship.

The class of 4-amino-2-sulfanylphenols is obviously an outlier among the reviewed compounds, as the 2-sulfanylphenol substructure is not a structural element in natural products. The vast majority of thiol groups in secondary metabolites derive from the amino acid cysteine, producing aliphatic secondary metabolites with thiol functionalities instead of phenolic ones. Nevertheless, this class contains thio-analogs of the naturally occurring *o*-catechol substructure, and thus, it has been included in this review. Zhang and Xu et al. [51] have reported that 4-amino-2-sulfanylphenol derivatives display highly specific protein kinase and angiogenesis inhibitory activities. Based on their previous findings, the structure of compound **24** was optimized by replacing the naphthalene by a phenolic skeleton and a sulfonamide fragment [52]. These compounds show in vitro anti-angiogenic activity with respect to Pazopanib in both HUVEC tube formation assay and the rat thoracic aorta ring test. They inhibited protein kinase B/Akt and ABL tyrosine kinase in the micromolar range. The preliminary structure–activity relationship is summarized in Figure 5.

**Scheme 6:**
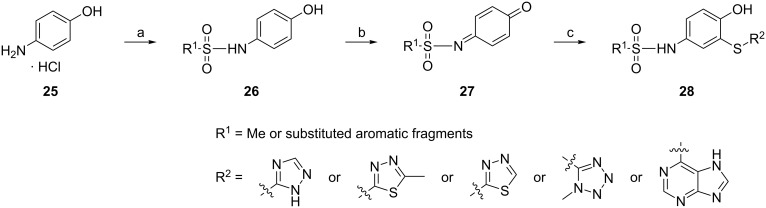
Synthesis of 4-amino-2-sulfanylphenol derivatives. Reagents and conditions: (a) R^1^SO_2_Cl, pyridine, 0 °C; (b) NaIO_4_/SiO_2_, DCM; (c) DMF, R^2^SH.

The synthesis of 4-amino-2-sulfanylphenol compounds (Scheme 6) [53] starts from the 4-aminophenol hydrochloride salt (**25**), which reacted with various substituted sulfonyl chlorides in pyridine, yielding compound **26** that differs in the substituent R^1^. Oxidation by NaIO_4_/SiO_2_ gives the quinone-type structures **27**, which were reacted without further purification with R^2^SH thiols. The addition to the unsaturated system yielded the target compound **28** with different arylthiol groups under rearomatization.

### Acylphloroglucinol derivatives

**Figure 6:**
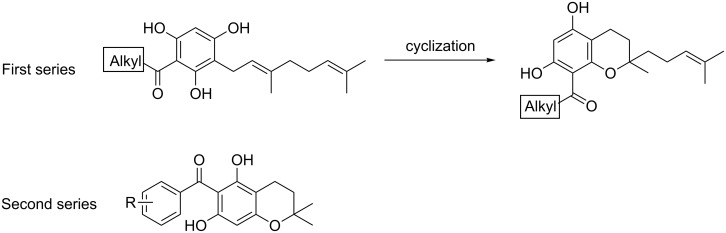
Structures of two series of natural-like acylphloroglucinols.

Acylphloroglucinols are typical secondary metabolites biosynthetically derived from the polyketide pathway and mainly accumulate in Hypericaceae [54] and Clusiaceae. Hyperforin, likely the most prominent acylphloroglucinol derivative and present in higher concentration in St. John’s wort (*Hypericum perforatum* L., Hypericaceae), has been recently reported to exhibit strong antiproliferative effects [55] that strongly inhibit angiogenesis in both in vitro and in vivo models. Mechanistically, it interferes with MMP-2 and a urokinase plasminogen activator (uPA) [56]. However, due to its instability in aqueous solution, complex structure and limited availability, hyperforin is neither a drug candidate nor a good model compound. The finding that structurally simpler, natural, acylphloroglucinol derivatives also showed antiproliferative effects against endothelial cells with inhibitory effects in a tube-formation assay on Matrigel catalyzed the search for simple acylphloroglucinols with anti-angiogenic activity. Within this group, some geranylated monocyclic and bicyclic acylphloroglucinol derivatives have been identified that are structurally much more simple than hyperforin. However, they exhibiting potent antiproliferative activity for a human microvascular endothelial cell line (HMEC-1) at low micromolar concentrations [57–58]. Two series of natural-like acylphloroglucinols were synthesized (Figure 6) for the systematic investigation of their anti-angiogenic properties [59].

**Scheme 7:**
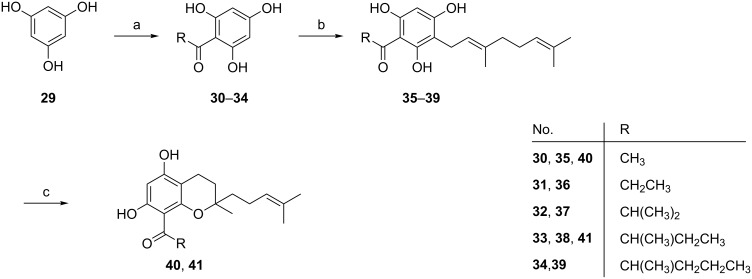
Synthesis of acylphloroglucinol derivatives **35–41**. Reagents and conditions: (**a**) acyl chloride, AlCl_3_, CS_2_/PhNO_2_, 55 °, 2 h; (**b**) geranyl bromide, K_2_CO_3_, acetone, reflux, overnight; (**c**) pTSA, benzene, reflux, 2 h.

The compounds of the first series [59] were prepared starting from 1,3,5-trihydroxybenzene (**29**, Scheme 7). Compounds **30–34** were obtained through Friedel–Crafts acylation of **29** with corresponding acyl chlorides in 55–81% yield. Subsequent alkylation with geranyl bromide gave products **35–39** in moderate yields from 55 to 60%. Finally, *p*-toluenesulfonic acid (pTSA)-catalyzed cyclization afforded the target compounds **40** and **41** in 53 and 65% yield, respectively.

**Scheme 8:**
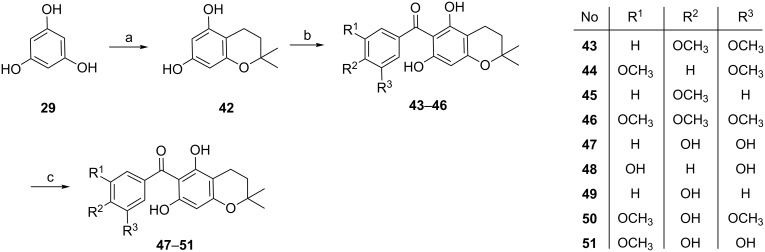
Synthesis of acylphloroglucinol derivatives **43–51**. Reagents and conditions: (**a**) isoprene, Amberlyst 15, THF/hexane; (**b**) benzoyl chloride, AlCl_3_, DCM, −5 °C to rt, overnight; (**c**) BBr_3_, DCM, −78 °C to rt, overnight.

The synthesis of the second series is shown in Scheme 8 [59]. Amberlyst 15 efficiently catalyzed the condensation of 1,3,5-trihydroxybenzene (**29**) with isoprene in 53% yield. The following Friedel–Crafts acylation gave the intermediates **43–46**, which were subsequently demethylated using BBr_3_ to give compounds **47–51** in 48 to 78% yield.

Compound **47** (R^1^ = H, R^2^ = OH, R^3^ = OH) showed antiproliferative activity with an IC_50_ of 0.88 ± 0.08 µM in vitro*.* Compound **38** (alkyl = CH(CH_3_)CH_2_CH_3_) exhibited a moderate antiproliferative effect (IC_50_ = 11.0 ± 1 µM) and inhibited the capillary-like tube formation of HMEC-1 in vitro, whereas **47** was inactive. Furthermore, some of the compounds showed significant antioxidative activity. The most active compound in the ORAC assay was compound **47**, which exhibited an antioxidative effect of 6.6 ± 1.0 TE. However, this compound showed only weak activity during the proliferation assay (IC_50_ = 53.8 ± 0.3 µM) and did not inhibit tube formation.

A basic structure–activity relationship of the aliphatic mono- and bicyclic acylphloroglucinol derivatives with short acyl side chains indicates that the in vitro antiproliferative activity of these acylphloroglucinols in HMEC-1 increases with increasing log P. Increasing the number of carbon atoms in the acyl group provides higher lipophilicity, which allows the compound to better penetrate across cellular membranes in the in vitro assay. In contrast, the activity of the derivatives with aromatic acyl side chains depends on other properties. The molecular aspects of the observed cellular effects are currently under investigation.

### (−)-Epigallocatechin-3-*O*-gallate (EGCG)

**Figure 7:**
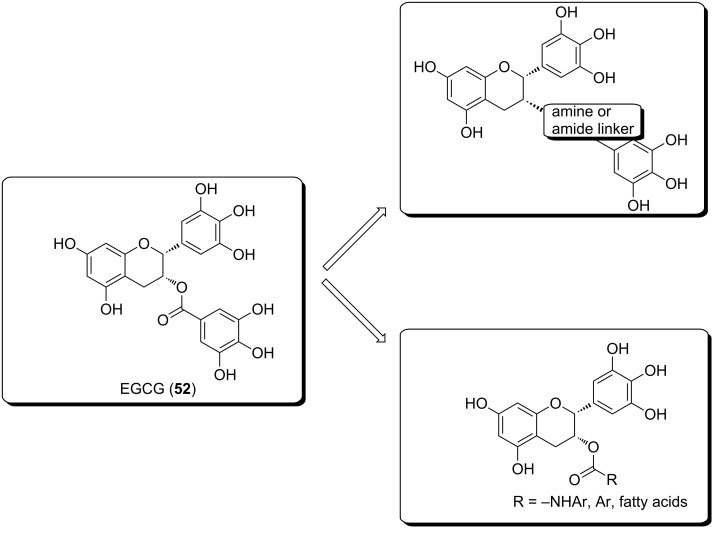
Analogs of (−)-EGCG for the prevention of oxidation and improvement of the bioavailability of the compounds [67,70–72].

(−)-Epigallocatechin-3-*O*-gallate (EGCG, **52**) is the most abundant catechin in green tee (*Camellia sinensis* L. KUNZE, Theaceae). It is the esterification product of epigallocatechin and gallic acid. Many studies provide evidence that EGCG modulates multiple signal transduction pathways, controlling the unwanted proliferation of cells. It inhibits the activation of HIF-1α, NF-κB and VEGF expression, thereby suppressing tumor angiogenesis and cancer progression [60–61]. Furthermore, EGCG inhibited MMP-2 and MMP-9 (in different cell types), which seem to play an important role in tumor invasion and metastasis. Also, the inhibition of uPA by EGCG has been observed [62]. Urokinase plasminogen activator (uPA) has the ability to prevent apoptosis, it also stimulates angiogenesis, mitogenesis, cell migration, as well as modulates cell adhesion. The presence of the 3-galloyl moiety in catechins led to higher biological activity [63–65], but an increasing number of aromatic hydroxy groups result in low stability and the inability of the compound to cross cellular membranes [66]. To prevent oxidation and improve its bioavailability, modifications of EGCG focus on synthesizing more stable analogs (Figure 7). Anderson et al. [67] replaced the hydrolytically labile ester bond with a more stable amine and amide bond and evaluated their efficacy as modulators for β-lactam resistance in *S*. *aureus*. Landis-Piwowar et al. [68] and Wang et al. [69] protected the hydroxy groups through peracetylation. These analogs behave as prodrugs and the acetyl group is removed by cellular cytosolic esterases. Liao et al. [70] and Huang et al. [71] acylated the phenol group at the 3-position, introduced fatty acids of different sizes and extensively explored their structure–activity relationship to 5α-reductase. Park and co-workers [72] also synthesized 3-*O*-alkyl analogs of epicatechin. They found that the introduction of alkyl groups instead of acyl groups enhanced the antimicrobial activity and stability at pH 7.4.

### Xanthohumol

**Scheme 9:**
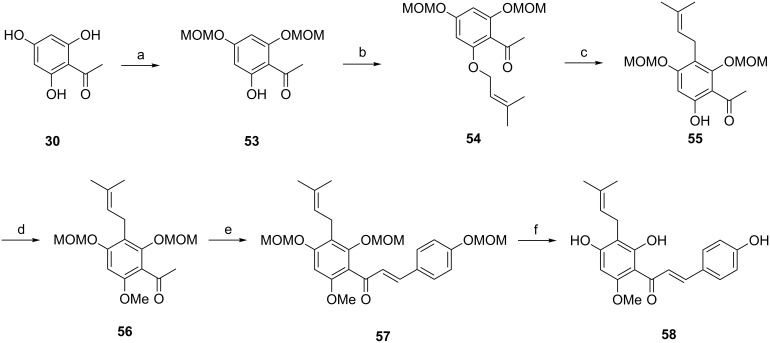
Synthesis of xanthohumol **58**. Reagents and conditions: (a) MOMCl, diisopropylethylamine, CH_2_Cl_2_; (b) 3-methyl-2-butene-1-ol, diethyl azodicarboxylate, PPh_3_, toluene/THF; (c) *N*,*N*-dimethylaniline, reflux; (d) (CH_3_O)SO_2_, K_2_CO_3_, acetone, reflux; (e) 4-methoxymethylbenzaldehyde, aqueous NaOH, MeOH, reflux; (f) concentrated HCl (pH 1), MeOH/H_2_O, rt.

Xanthohumol (XN, **58**), a naturally occurring prenylated chalcone in hop plants (*Humulus lupulus* L., Cannabaceae), has been suggested to have potential to suppress the development and progression of cancer and is therefore also a compound of chemopreventive potential [73–74]. For example, XN shows proliferative inhibition of human breast (MCF-7), colon (HT-29), and ovarian cancer (A-2780) cells in vitro with IC_50_s ranging from 0.52 to 13.3 μM. Because most cancer chemopreventive agents have also anti-angiogenic properties in vitro and in vivo, further investigations [75] showed that XN repressed both the NF-κB and Akt pathways in endothelial cells, inhibited VEGF-A expression in a wound-healing assay, and exhibited interference in the angiogenic process. The first complete synthesis of xanthohumol was accomplished by Erhardt et al. in six steps with an overall yield of 10% in 2007 (Scheme 9) [76]. The method was improved by Vogel and Heilmann et al. [77–78] to yield also several xanthohumol derivatives occurring as minor compounds in hop cones or as in vivo metabolites after xanthohumol intake [77]. To date, xanthohumol in vivo metabolites have not been investigated regarding their anti-angiogenic activity.

### Genistein

**Scheme 10:**
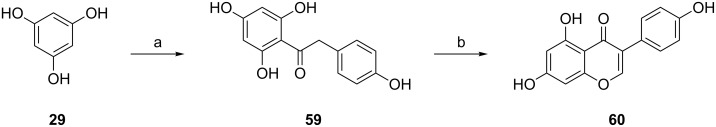
Synthesis of genistein **60**. Reagents and conditions: (a) 4-hydroxyphenylacetonitrile, anhydrous HCl, ZnCl_2_·Et_2_O, then aq HCl, heating or 4-hydroxyphenylacetic acid, BF_3_·Et_2_O, 120 °C; (b) BF_3_·Et_2_O, DMF, MeSO_2_Cl, 100 °C, 2 h.

Genistein (**60**) is an isoflavone extracted from soybeans (*Glycine max* (L.) MERR, Fabaceae). It is present as the 7-*O*-glycoside genistein in the plant; however, during the processing of soya products, a significant amount of the aglycone genistein is released. Genistein was originally described as an exclusive inhibitor of tyrosine-specific protein kinases [79]. These kinases are responsible for the tyrosine-specific protein phosphorylation, which is required for the regulation of cell functions, including cell proliferation and cell transformation. Later, genistein was also found to act as an oestrogen receptor agonist [80]. The anti-angiogenic potential of genistein was first reported by Fotsis et al. in 1993 [81]. Then, further studies showed that genistein inhibited angiogenic processes in various in vitro and in vivo models [82–83]. The typical synthesis of genistein starts from 1,3,5-trihydroxybenzene (**29**, Scheme 10) [84]. After the Houben–Hoesch reaction or Friedel–Crafts acylation, the cyclization of the resulting hydroxyketone **59** in the presence of BF_3_·Et_2_O gave genistein (**60**) in good yield.

In the Friedel–Crafts acylation, BF_3_·Et_2_O was used as the catalyst and solvent. The following formation of the pyrone was also catalyzed by BF_3_·Et_2_O, and a convenient one-pot synthesis of **60** was achieved without isolation of **59** [85].

### Fisetin and quercetin

The flavonoids fisetin (**67**) and quercetin (**68**) belong to the flavonol subgroup that exhibit a double bond between C-2/C-3 and a hydroxy group at C-3. Flavonols are the most abundant flavonoid subtype in plants and commonly occur as glycosides. Nevertheless, pharmacological testing was historically concentrated on the investigation of the aglycones. Although this has been often criticized, it is most likely an important aspect with regard to flavonoid metabolism. It has been shown that the flavonoid glycosides are not absorbed after oral intake but are cleaved by lactase-phlorizin hydrolase and absorbed as the corresponding aglycone. The aglycone passes through the cell membrane by passive diffusion and undergoes phase-II metabolism in enterocytes and the liver, leading to glucuronides as the main metabolites. However, a release of the aglycones from the glucuronides in tissues or cells with β-glucuronidase activity is possible [86].

Fisetin can be found in many fruits such as strawberries and apples (*Malus* spec. MILL., Rosaceae) as well as in vegetables such as onions (*Allium cepa* L., Amaryllidaceae, subfamily Allioideae former family Alliaceae). It shows anticancer activity in various cancer models. For example, it can inhibit androgen receptor signaling and tumor growth in athymic nude mice [87], it can cause apoptosis and cell-cycle arrest in human prostate cancer LNCaP cells [88] and in HCT-116 human colon cancer cells, and it can induce apoptosis associated with an increased level of p53 [89]. Singh and Bhat et al. [90] tested its anti-angiogenic activity for the first time. Their study revealed that fisetin (10–50 µM) strongly inhibits the growth, proliferation and cell-cycle progression in HUVECs by downregulating the expression of VEGF and eNOS. Another recent study [91] also demonstrated that fisetin inhibits MMPs and reduces tumor-cell invasiveness and endothelial cell tube formation.

Together with kaempferol, quercetin is the most abundant aglycone in flavonol glycosides. Quercetin glycosides occur in higher concentrations in onions, red wine, and green tea and in various medicinal plants [92]. In a number of early studies [93–94], quercetin showed a strong ability to inhibit tumor growth in vivo. Quercetin suppresses angiogenesis through multiple mechanisms such as inhibition of COX-2 and lipoxygenase (LOX)-5, interference with the EGF receptor, the human epidermal growth factor receptor-2 (HER-2) intracellular signaling pathway, and the NF-κB nuclear transcription protein [7–8]. Chen et al. [95] reported that quercetin inhibited the proliferation of choroids retina endothelial cells and the migration and tube formation of RA/6A cells were also significantly inhibited by quercetin in a dose-dependent manner. In some cell types, quercetin is also able to activate the angiogenic pathway by inhibiting HIF-prolyl hydroxylase [96]. Zhao et al. [97] investigated the anti-angiogenic activity of quercetin in transgenic zebrafish embryos and in HUVECs. The formation of intersegmental vessels was disrupted in the zebrafish model. In HUVECs, quercetin inhibited cell viability, the expression of VEGF-2 and tube formation in a dose-dependent manner.

**Scheme 11:**
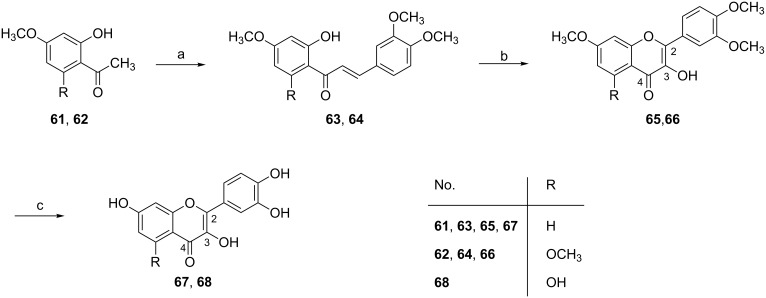
Synthesis of fisetin (**67**) and quercetin (**68**). Reagents and conditions: (a) 3,4-dimethoxybenzaldehyde, KOH, DMF, 0 °C; (b) BiCO_3_, AcOH, 2-ethoxyethanol, Δ; (c) BBr_3_, DCM, −78 °C → rt.

The synthesis of fisetin and quercetin can be achieved by two different methods. The first choice for synthesizing fisetin and quecetin is by the Allan–Robinson reaction [98]. However, this reaction has some drawbacks such as harsh experimental conditions and the necessity of selective protection and deprotection of the hydroxy groups with benzyl and/or benzoyl groups. An alternative method is the Algar–Flynn–Oyamada (AFO) reaction [99]. This allows the direct synthesis of flavone-3-ols, although the yields vary depending on the substrates used. Simpson et al. [100] improved the reaction conditions of AFO in order to synthesize the flavonol rhamnocitrin by using bismuth carbonate and acetic acid. This increased the yields to 71% and the overall yields to 52% over two steps. According to this, a synthesis of fisetin and quercetin with methyl-protected chalcones as starting materials was proposed (Scheme 11).

### (2*S*)-2’,4’,7-Trihydroxy-5-methoxy-8-(dimethylallyl)flavanone

**Figure 8:**
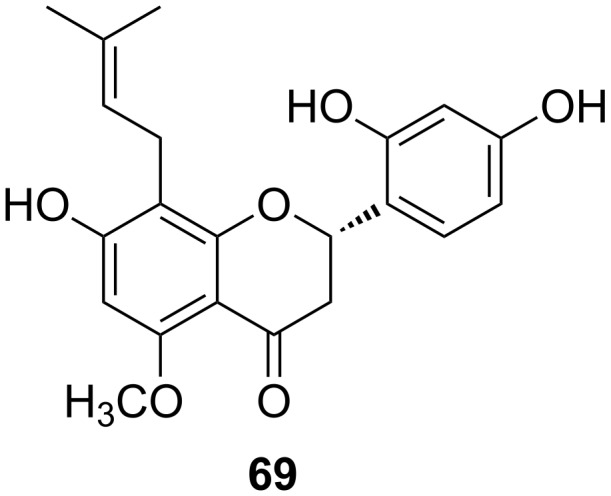
Structure of (2*S*)-7,2’,4’-trihydroxy-5-methoxy-8-(dimethylallyl)flavanone (**69**).

(2*S*)-7,2’,4’-Trihydroxy-5-methoxy-8-(dimethylallyl)flavanone (**69**, Figure 8) is a prenylated flavanone isolated from *Sophora flavescens* by Wang and Yuan et al. in 2013 [101]. It displays inhibitory effects on cell proliferation, cell migration, cell adhesion and tube formation in the human umbilical vein endothelial cell line ECV304. These four steps are important in angiogenesis. A mechanistic study showed that compound **69** is able to downregulate ROS levels and VEGF expression in a dose-dependent manner, and to arrest cell cycle in the G0/G1 phase.

## Conclusion

As pathological angiogenic processes are believed to contribute to several diseases, compounds with anti-angiogenic activity have been intensively investigated. In addition to antibodies, several low molecular weight compounds have also been chemically and pharmacologically characterized and among them, several secondary metabolites of natural origin. In anti-angiogenic strategies, natural products with phenolic substructures or belonging to polyphenols are of special importance as they occur in several food and medicinal plants important for human diet and health. Despite the fact that phenolic compounds often show specific interactions in biological systems, most of them are pleiotropic substances with effects on different cellular networks or targets. Several natural phenolic angiogenic inhibitors such as curcumin (**3**), epigallocatechin-3-*O*-gallate (**52**) and xanthohumol (**58**) also showed remarkable chemopreventive activity. However, the stability, availability from natural sources and bioactivity of the natural compounds are typically limited. Thus far, no example of very strong anti-angiogenic activity in the nanomolar range has been found. Thus, synthetic approaches for the production, diversification and optimization are required. The modification of the polyphenol structures to improve their stability and bioactivity and further enhance their anti-angiogenic activity is the main goal of current research in the field. Analogs of the natural phenols with improved drug properties may be promising candidates for future oncology treatment.
